# Detection of Epithelial Ovarian Cancer using C8Magnetic Bead Separation and MALDI-TOF Plasma Proteome Profiling in Egyptian Females

**DOI:** 10.31557/APJCP.2019.20.12.3603

**Published:** 2019

**Authors:** Mohamed Mostafa Rizk, Ola Atef Sharaki, Mahmoud Elsayed Meleis, Doreen Nazeih Younan, Alyaa Abdallah Elkayal, Pacinte Moez

**Affiliations:** 1 *Department of Clinical Pathology, *; 2 *Department of Obstetrics and Gynecology, Faculty of Medicine, University of Alexandria, Egypt. *

**Keywords:** Carcinoma, ovarian, epithelial, mass spectrometry, proteome

## Abstract

**Background:**

Ovarian cancer is the seventh most common cancer in females with the highest mortality rate of all gynecological cancers due to its late discovery and ambiguous symptoms. Thus, there is a need for new promising strategies to diagnose ovarian cancer. We aimed at finding a characteristic plasma proteome pattern that could be used for the detection of epithelial ovarian cancer, in comparison with benign ovarian masses and healthy controls. We also aimed at differentiating between profiling of plasma proteins in early and advanced stages of ovarian cancer and between serous and non-serous histopathological types.

**Methods:**

The combination of MagSi-proteomics C8 beads, Ultraflextreme MALDI-TOF and ClinPro Tools software was used to compare the plasma protein spectra from 50 patients with epithelial ovarian cancer, 20 patients with benign ovarian masses and 50 age matched healthy females.

**Results:**

A plasma proteome profile of 21 peaks differentiated patients with epithelial ovarian cancer from healthy controls with a sensitivity of 73 % and a specificity of 82.8% upon external validation, while a 5-peak profile differentiated patients with epithelial ovarian cancer from patients with benign ovarian masses with a sensitivity of 81% and a specificity of 73.7%. A 20 peak profile was generated to discriminate between early and late stages of the disease with 88.3% recognition capability and 70% cross validation.

**Conclusion:**

MALDI-TOF proteomic profiling represents a promising potential tool for diagnosing epithelial ovarian cancer, discriminating between early and advanced stages and between serous and non- serous types.

## Introduction

Gynecologic cancers constitute a major health burden in Egypt. Ovarian cancer is the 4^th^ commonest cancer among Egyptian females (Ibrahim et al., 2014). It has the highest mortality rate of all gynecological cancers (Ferlay et al., 2015). Epithelial ovarian cancer represents the most common histopathological subtype; counting for 90% of all ovarian cancers (Sedláková et al., 2015). The prognosis for ovarian cancer patients is poor, particularly when diagnosed in its advanced stages (Heintz et al., 2006). Symptoms are ambiguous, often misdiagnosed and so, current diagnostic tools have very limited success in early detection (Goff et al., 2000; Goff et al., 2004). As a result, the majority of patients are only identified in the advanced stages of the disease (Heintz et al., 2006).

High-grade ovarian cancer (HGOC) is the leading cause of mortality from gynecological malignancies, because of diagnosis at a metastatic stage. The 5-year-survival rate for patients in stage III is reported to be 35% while in stage IV is as low as 22%. Current screening options fail to improve mortality because of the absence of early-stage-specific biomarkers (Barnabas et al., 2019).

The tumor marker CA125, initially described by Bast et al., is still widely used for the routine diagnosis of adnexal masses. It is also used for monitoring the response to treatment, follow-up of the disease, and detection of disease recurrence. However, this tumor marker can be increased in several gynecological and non-gynecological diseases, and this reduces the diagnostic accuracy for the detection of ovarian cancer (Hogendorf et al., 2017). 

Several different mathematical models and scoring systems have been created, based on clinical features, ultrasound findings, and/or serum level of tumor markers, aimed at increasing the diagnostic performance of each individual parameter. One such model is the Risk of Ovarian Malignancy Algorithm (ROMA) created by Moore et al., (2010). The ROMA combines the tumor markers CA125 and HE4 using two formulas, taking into account the menopausal status of each patient (Abdalla et al., 2016).

In 2009, the FDA approved the OVA1 test to assess preoperatively the risk of ovarian cancer among women with pelvic masses (Toss et al., 2013). The OVA1 test comprises the following five proteomic biomarkers: CA125, transthyretin (prealbumin), apolipoprotein A1, beta 2 microglobulin and transferrin (Rai et al., 2002; Zhang and Chan, 2010; Fung, 2010).

While molecular assays based on mutation detection are challenged by the rarity of tumor DNA within non-mutated DNA, analyzing the proteomic profile, is expected to enable earlier detection, as it reveals abnormalities in both the tumor as well as in its microenvironment (Barnabas et al., 2019).

The study of the ovarian proteomic profile represents a new approach in ovarian cancer research. Due to the possibility of analyzing thousands of proteins, which could be simultaneously altered, comparative proteomics represent a promising model of possible biomarker discovery for ovarian cancer detection and monitoring. Moreover, ongoing research studies, that define signaling pathways in ovarian cancer cells through proteomic analysis, offer the opportunity to design novel drugs and to optimize the use of molecularly targeted agents against crucial and biologically active pathways. Proteomic techniques provide comprehensive information about different histological types of ovarian cancer, cell growth and specific molecular targets predictive of response to chemotherapy (Toss et al., 2013).

Given the relatively high prevalence of ovarian cancer, came out the need for strategies with high sensitivity and specificity for its detection, especially in early stage (Badgwell et al., 2007). CA125, which is the most commonly used serum molecule in the clinical practice (Kobayashi et al., 2012), even when integrated with trans-vaginal sonography, both can only detect about 25% of ovarian cancers in the early stage (Fan et al., 2010). There are various types of novel ovarian cancer markers investigated at present; of these, the protein-based ovarian cancer biomarkers. With the greatest advances in proteomic technologies, we can explore the low molecular part of the plasma proteome for tumor markers through the use of mass spectrometry (MS) to detect this portion of the proteome. It is unlikely that a single biomarker will detect all subtypes and stages of ovarian cancer due to its complexity and heterogeneity. Combining several biomarkers dramatically improves the sensitivity of detection methods in ovarian cancer patients (Yurkovetsky et al., 2006; Zhang et al., 2011). The study of ovarian cancer proteomic profile using the combination of magnetic beads and MALDI-TOF-MS is assumed to be a key role in most proteomic workflows (Diamandis, 2004).

The optimum diagnosis of the malignant status of masses is important as it facilitates the selection of patients with malignant masses who need urgent referral to gynecological oncology centers and consequently improves the overall survival rate for patients with ovarian cancer (Anderson et al., 2019).

In this study we aimed at assessing the role of MALDI-TOF MS in studying the plasma proteome profile of patients with epithelial ovarian cancer, in comparison to healthy controls and to those having benign ovarian lesions. Our goal was to detect a disease-specific pattern that can be validated for detection of epithelial ovarian cancer, thus, providing a useful screening and diagnostic tool for such a disease. We also aimed at differentiating between profiling of plasma proteins in early and advanced stages of ovarian cancer and between serous and non-serous histopathological types.

## Materials and Methods

*Study Design*

A prospective, case control study.

*Subjects*

Fasting blood samples were withdrawn from 120 subjects: 50 patients newly diagnosed with epithelial ovarian cancer (group I), 20 patients with benign ovarian masses (group II) and 50 age matched healthy females, served as a control group (group III), who had no history of gynecologic tumors and had normal pelvic examination and/or pelvic ultrasonography. All patients were recruited from the Gynecology Department, Faculty of Medicine, Alexandria University Teaching Hospital in Egypt, over a period of 8 months. 

This study received ethical approval from the Institutional Research Ethics Committee at Faculty of Medicine, Alexandria University. Written informed consents were obtained from all participants before enrollment in the study. The identification information of all subjects was kept confidential and was protected from the public. This work has been carried out in accordance with the Code of Ethics of the World Medical Association (Declaration of Helsinki and its later amendments) for experiments involving humans.

Epithelial ovarian cancer was diagnosed by histopathological examination of patients’ surgical specimens. According to their histopathology, 27 patients had serous cystadenocarcinoma, 11 patients had endometroid adenocarcinoma, 8 patients had mucinous cystadenocarcinoma and 4 had other subtypes. According to FIGO staging; 12 cases were stage I, 17 were stage II, 19 were stage III and 2 were stage IV.

*Methods*

- History taking, physical examination and radiological investigations were done to all patients, to determine the extent of the disease and tumor stage in group I.

- Routine and specific laboratory investigations were also done (pre-operatively, for those requiring surgery for ovarian tumors); including serum CA-125 and CEA levels, that were measured by electro-chemiluminescence, using Advia Centaur XP automated immunoassay analyzer (Siemens Healthcare Diagnostics, USA).

- Histopathological examination of the surgical specimen was done to tumors excised from groups I and II, to determine tumor grade and extent of invasion of nearby tissues/organs; uterus, omentum, colon or urinary bladder, in group I.

- Risk of malignancy index 2 (RMI2) was calculated using the following formula; (Yenen et al., 2012)

RMI2 = ultrasound score x menopausal score x CA-125 level in U/mL.

- Plasma Proteomic Profiling using C8 Magnetic Beads and Ultraflextreme MALDI-TOF Mass Spectrometer: (Fan et al., 2010)

*Sample Processing*

Blood samples for proteomic profiling were aseptically collected, pre-operatively, into K2-EDTA vaccutainer tubes. The tubes were centrifuged at 1800g at 4oC for 15 minutes. Then plasma samples were aliquoted and frozen at -800C, within 2 hours, till further use.

*Protein Separation*

For protein separation, samples were thawed at room temperature and processed immediately. The low- molecular-weight peptides were separated from the plasma using MagSi-Proteomics C8 beads (Magna Medics). They are magnetic silica beads coated with C8 alkyl groups providing a reversed phase surface chemistry. Peptides and proteins were separated by binding to the hydrophobic surface of the beads. After elution of the bound proteins/peptides, 1 µL of the elute was spotted on MTP polished steel target and left to dry at room temperature. Then 1 µL of α-cyano-4-hydroxycinnamic acid (HCCA) matrix was applied then left to dry at roomtemperature.2-4 spots were spotted on the target for each sample.

*MALDI-TOF MS Spectra Acquisition*

FlexControlTM software allows using the UltrafleXtreme MS for laser shooting of the samples and acquisition of the spectra for proteomic analysis. Spectra acquisition was done using the positive linear mode of the MALDI-TOF/ UltrafleXtreme MS (Bruker Daltonics), with the following settings: ion source 1: 25 kV; ion source 2: 23.45kV; lens: 6 kV; pulsed ion extraction: 100 ns. Mass calibration was performed using the ClinProt standard (CPS), as a standard sample. From the FlexControlTM software, we set the detection limit of the spectrometer to 800-20000 Da and then 3000 laser shots were done by shooting 500 laser shots at 6 different positions in each target plate spot. After finishing the shooting of spots of each sample, we gathered them into one spectrum using the sum buffer icon in the toolbar and then adjusted using baseline subtraction and spectral smoothing. Only peaks with signal/noise ratio (S/N) above 3 were chosen for better spectral resolution. For each subject, spectra from 2-4 MALDI spots were generated.

*Analysis using ClinPro Tools Software*

The spectra of all signals were analyzed using ClinPro Tools V.03software to generate the proteomic profiles. Each group has been randomly distributed into two groups; training set for model generation, and validation set for external validation.

To differentiate between plasma proteome profiles of groups I and III, each group was randomly split into 2 sets; a training set (25 subjects) and a validation set (25 subjects). For differentiating between plasma proteome profiles of groups I and II, a training set was composed of 14 and 10 profiles respectively and validation set was composed of another 11 and 10 profiles. The mean value of peak intensity, SD and CV (%) for each corresponding peak were calculated. ClinPro Tools offers various statistical tests; Wilcoxon/ Kruskal-Wallis test (p<0.05) was used in our research to define significant difference.

For model generation, three classification algorithms were used: Genetic Algorithm (GA), Supervised Neural Network (SNN) and Quick Classifier (QC).

Cross validation, a measure of the reliability of a model was performed to predict the future performance of the model. It is done by splitting a certain data set into two sets: model generation set and test set. A model is generated from the model generation set and then the test set is used to evaluate this model and to determine the prediction capability. This procedure is repeated multiple times and the absolute prediction capabilities are accumulated and finally normalized to a relative prediction capability obtained by the cross validation procedure. Three procedures for cross validation are present in ClinPro Tools: Random, K-Fold and leave one out. The latter was used in our study, in which one data point was left out. The remaining points were used for model generation. The omitted data point was classified against the model. This procedure is repeated for n times, where n is the number of data points. The obtained classification results are stored for the n models, averaged and returned as the prediction capability.

Like cross validation, external validation was also carried out to predict the capability of the model. In external validation, none of the spectra used for model generation was used for validation. Only new set of spectra were loaded and used for external validation.

Specificity was calculated as the ratio of the number of negative samples correctly classified to the total number of true negative samples. Sensitivity was calculated as the ratio of the number of correctly classified diseased samples to the total number of diseased samples.

## Results

Group I included 50 patients newly diagnosed with epithelial ovarian cancer, with no prior therapy or intervention, whose main complaint was pelvic pain and mostly presenting with ascitis and pelvi-abdominal masses. 28% of them had positive family history for cancer (in general). Histopathologically, 54% of them were having ovarian cancer of the serous type, while the rest were non serous; either mucinous or endometroid. 58% (29/50) presented in an early stage of the disease (24% stage I & 34% stage II), while 42% (21/50) were in a late stage (38% stage III and 4% stage IV).

84% of group I (42/50), 90% of group II (18/20) and 74% of group III (37/50) were post-menopausal, with no significant difference (p=0.233). The three groups, included in this study, were matched for age; the median age was 56 years for both groups I and II and 54 years for group III (p=0.218). 

In group I, CA125 mean level ± SD was 596.77 ± 845.12 U/mL, however, it was much lower in groups II and III (63.02 ± 125.34 U/mL and 11.39 ± 5.04 U/mL respectively). As regards the mean CEA level ± SD, it was 5.97± 8.24 ng/mL in group I, 2.24 ± 1.03 ng/mL in group II and 1.84 ng/mL ± 1.03 in group III. Serum CA125 and CEA levels were significantly higher in group I, when compared to the other 2 groups, p=<0.001 for both (Cut off limit for CA125= 35 U/mL and for CEA=5 ng/mL).

*Plasma proteomic analysis of patients with epithelial ovarian cancer and healthy subjects*

In our study, 14 peaks differed significantly between groups I and III; 13 peaks of them were over-expressed in patients with ovarian cancer (group I), while 1 peak was under-expressed, as shown in Table 1. We used the SNN classification algorithm to build the differentiating model between cancer patients and controls, with the following settings: 6 used as the limit of signal/noise ratio (S/N ratio), Data Reduction Factor 4 and Noise Threshold 1. 21 peaks represented the proteomic profile that differentiates between both groups. These 21 peaks had m/z ratios as shown in Table 2. Besides their multivariate discriminatory power as a profile, 7 peaks of them with m/z ratios: 2504.22, 3856.81, 4676.84, 4789.57, 4813.94, 5816.49 and 5840.28 were significantly differentially expressed between the two groups; all were over-expressed in cancer patients. Their p values (PWKW) were 0.000314, 0.000433, 0.000433, 0.000759, 0.00464, 0.0216 and 0.0248 respectively. This proteomic profile identified 83.3 % of the patients’ spectra and 82.8% of the controls’ spectra during cross validation with a recognition capability of 89.4%. Upon external validation, a sensitivity of 73% and specificity of 82.8% were achieved.

*Plasma proteomic analysis of patients with epithelial ovarian cancer and those with benign ovarian masses*

Our study identified 31 peaks that showed significant difference between group I and group II. 20 peaks of them were over expressed in cancer patients, while 11 peaks were under expressed, as shown in Table 3.

We used GA classification algorithm in generating a model to discriminate between both groups with the following settings: 6 used as the limit of signal/noise ratio (S/N ratio), Data Reduction Factor 4 and Noise Threshold 1. In GA algorithm, unlike SNN or QC, the number of peaks constituting the model was set manually to 5 in order to obtain the best combination of 5 peaks as a discriminatory profile. These peaks had the following m/z ratios: 1,082.58, 1,277.23, 4,514.18, 6,432.14 and 8,809.95, as shown in Table 4. Besides their multivariate discriminatory power as a profile, the first 3 peaks with m/z ratios: 1,082.58, 1,277.23 and 4,514.18 were significantly differentially expressed between both groups; all were under-expressed in cancer patients. Their p values (PWKW) were 0.00307, 0.00613 and 0.00574 respectively. This proteomic profile identified 93.1% of group I spectra and 81.6 % of the group II spectra during cross validation with a recognition capability of 97.4%. Upon external validation, a sensitivity of 81% and a specificity of 73.7% were achieved.

*Plasma proteomic analysis of patients with epithelial ovarian cancer according to tumor stage*

Using the SNN model, a profile of 20 peaks was generated to differentiate patients with early ovarian cancer (58% of group I, 29/50) from those with late stages of the disease (42% of group I, 21/50), with a recognition capability of 88.3% and an overall cross validation of 70%. Of these 20 peaks, 14 were over-expressed in early stage ovarian cancer patients (stages I and II), but not significantly. Their m/z was 3475.91, 3,823.41, 4,789.96, 5,763.35, 5,794.77, 5,842.16, 5,863.09, 5,870.21, 8,765.79, 8,916.15, 9,131.72, 9,420.8, 11,684.09 and 11,528.53. While, 6 peaks with m/z 928.18, 1,044.24, 1,050.43, 1,060.53, 1,482.23, and 1,6610.96 were over-expressed in late stage ovarian cancer (stages III and IV). 

*Plasma proteomic analysis of patients with epithelial ovarian cancer according to tumor histopathological findings*

Using the GA classification algorithm, a 5-peak model was generated with the following m/z: 1,050.54, 2,210.89, 4,812.8, 8,739.46 and 11,318.04, to differentiate serous ovarian cancer from other non-serous histopathological subtypes (mucinous and endometroid). 3 of these peaks with m/z: 1,050.54, 2,210.89 and 4,812.8 were over-expressed in serous ovarian cancer in comparison to non-serous types. One peak with m/z 11,318.04 was under-expressed. Peak with m/z 8,739.46 was equally expressed in both serous and non-serous subtypes. This model reached a recognition capability of 84.5% and an overall cross validation of 74.9%. 

Moreover, 11 peaks were significantly expressed, from the univariate point of view, of which 5 peaks with m/z: 4,567.59, 4,677.25, 4,692.06, 6,525.87 and 6,714.31 were over-expressed in serous type and 6 peaks with m/z: 8,308.27, 9,511.03, 11,096.03, 11,318.04, 13,314.28 and 13,606.65 were over-expressed in non-serous types.

**Table 1:** The 14 Peaks which Were Significantly Expressed between Groups I and III (arranged in an ascending order according to mass).

Mass	PWKW^1^	Mass	PWKW^1^
2,094.37	0.047	5,704.13	0.00464
2,504.22	0.000314	5,735.58	0.00464
3,856.81	0.000759	5,816.49	0.000433
3,902.92	0.00832	5,840.28	0.000433
4,676.84	0.0248	5,863.89	0.00999
4,789.57	0.00464	8,764.54	0.000433
4,813.94	0.0216	11,681.13	0.0369

^1^PWKW, p value for Wilcoxon Kruskal Wallis; significant if < 0.05; - These PWKW values illustrate that, from a univariate point of view, these peaks can discriminate between patients with epithelial ovarian cancer and healthy controls; - The 13 highlighted peaks were over-expressed in group I, while only 1 peak was over-expressed in group III.

**Table 2:** The 21-peak Supervised Neural Network (SNN) Model Generated to Discriminate between Groups I and III

Index	Mass	Start mass	End mass
1	928.17	925.02	930.74
14	2,176.17	2,164.38	2,183.3
15	2210.71	2,202.31	2,222.76
17	2,504.22	2,495.89	2,516.22
18	2,754.75	2,746.04	2,765.84
19	2,939.07	2,929.95	2,949.62
20	3,086.21	3,074.64	3,096.40
26	3,475.57	3,465.36	3,484.18
29	3,822.97	3,811.16	3,833.6
30	3,856.81	3,848.00	3,866.94
38	4,676.84	4,663.82	4,684.67
39	4,690.73	4,684.67	4,696.61
41	4,789.57	4,765.57	4,799.73
42	4,813.94	4,799.73	4,817.86
49	5,816.49	5,803.83	5,823.78
50	5,840.28	5,823.78	5,853.77
56	6,630.54	6,601.43	6,646.39
54	6,477.60	6,474.46	6,490.85
57	6,714.70	6,706.98	6,726.05
77	8,915.35	8,891.39	8,931.21
79	9,131.08	9,069.21	9,194.31

- The 7 highlighted peaks were significantly over-expressed in ovarian cancer patients.

**Table 3:** The 31 Peaks which Were Significantly Expressed between Groups I and II (arranged in an ascending order according to mass).

Mass	PWKW^1^	Mass	PWKW^1^
1066.42	0.00289	4,812.76	0.000115
1082.58	0.00307	5,703.41	0.0266
1088.12	0.00574	5,735.10	0.0262
1104.95	0.0146	8,308.25	0.0107
1271.45	0.0364	9,492.55	0.000382
1277.23	0.00613	9,511.22	0.0000254
1293.51	0.0106	9,620.77	0.000185
1482.01	0.00662	11,074.26	0.0000248
1487.32	0.0262	11,095.25	0.0000248
1504.06	0.0241	12,360.38	0.000719
2094.19	0.0148	12,799.73	0.000763
2504.03	0.000163	13,288.21	0.0000248
3856.35	0.00655	13,313.53	0.0000254
4514.18	0.00574	16,606.55	0.0000248
4676.65	0.0146	16,636.01	0.000115
4789.22	0.00184		

^1^PWKW, p value for Wilcoxon Kruskal Wallis; significant if < 0.05; - These PWKW values illustrate that, from a univariate point of view, these peaks can discriminate between patients with epithelial ovarian cancer and those having benign ovarian masses; - The 20 highlighted peaks were over-expressed in group I, while the other 11 peaks were over-expressed in group II.

**Table 4:** The 5-peak Genetic Algorithm (GA) Model Generated to Discriminate between Groups I and II

Index	Mass	Start mass	End mass
6	1,082.58	1,078.08	1,086.64
11	1,277.23	1,273.28	1,285.18
40	4,514.18	4,502.25	4,523.72
56	6,432.14	6,407.62	6,446.08
75	8,809.95	8,777.59	8,823.99

- The 3 highlighted peaks were significantly under-expressed in ovarian cancer patients.

## Discussion

In this study, we introduced the use of MALDI–TOF-MS combined with magnetic beads and ClinPro Tools software for generation of proteomic profiles that could be used for diagnosis of epithelial ovarian cancer, and that could discriminate between early and advanced stages of the disease.

In our study, we identified a proteomic profile of 21 peaks that can discriminate between patients with epithelial ovarian cancer and healthy controls. Of these, peaks 3,475.57 and 2,939.07 could be related to peaks 3,470 and 2,937 respectively, in the proteomic profile identified by Fan et al., (2010), Peak 6,630.54, in our profile, could be related to peak 6646.1 identified by Timms et al., (2014) and to peak 6,635.1 identified by Qiu et al., (2009). Our results showed that a 5-peak profile could discriminate epithelial ovarian cancer from benign ovarian masses. Of these, peak 6,432 could be related to peak 6,440 identified by Wu et al., (2012). Regarding the proteomic profile generated to differentiate epithelial ovarian cancer patients according to tumor stage, Fan et al., (2010) reported over-expression of peak 3470 and peak 8775 in early stage ovarian cancer, which could be related to peak 3,475.91 and peak 8,765.79 in our profile, respectively. We also found these peaks to be over-expressed in early stages of the disease.

In our study, during cross validation between patients with epithelial ovarian cancer and healthy controls, 83.3 % of the patients’ spectra and 82.8% of the controls’ spectra were correctly identified. Upon external validation, a sensitivity of 73% and a specificity of 82.8% were achieved. Upon cross validation between patients with epithelial ovarian cancer and those with benign ovarian masses, 93.1% of the cancer patients’ spectra and 81.6 % of the benign masses patients’ spectra were correctly identified. Upon external validation, a sensitivity of 81% and a specificity of 73.7% were achieved. Wu et al., (2012) identified patients with ovarian cancer, during cross validation, with a calculated sensitivity of 90% and a specificity of 86.7%, which were comparable to our results. Upon external validation, they achieved a higher sensitivity of 88% and a specificity of 83.3%. 

We did not perform tandem MS, but we identified some peaks with reference to the published literature (Albrethsen, 2011). Some identified peaks corresponded to some newly discovered ovarian cancer biomarkers, such as peaks 11,684.09 and 11,528.53 that have been identified as serum amyloid A1 and were found to be up regulated in cancer patients in comparison to patients with benign masses. The same biomarker has been identified by Helleman et al., (2008) and Moshkovskii et al., (2005). Peaks with m/z 1,050.43 and 1,050.54, identified as fragments of complement C3F, have also been identified by Scholler et al., (2008). Peak with m/z 1,082.58 has been identified as fragment of fibrinopeptide-A. Bergen et al., (2003-2004) found several candidate biomarkers; one of them contained the sequence of fibrinopeptide-A.

Among the strong points in our study is the uniformity of the patients selected; all newly diagnosed with ovarian cancer with no prior therapy or intervention, age matched with patients having benign ovarian masses and with healthy controls. All parameters for plasma proteome analysis have been standardized starting from sample preparation and processing. Most studies for proteomic profiling in ovarian cancer were done using serum samples, however, in our study, we used plasma samples to seek novel candidate biomarkers that might differ from those in serum. Through our study, we were able to reach a discriminate protein signature pattern for epithelial ovarian cancer; whether early or late stage and also for benign ovarian masses. The identified protein peaks may be candidate proteins for early detection of ovarian cancer or evaluation of therapeutic responses.

Among the main limitations of our study, are the relatively small sample size and the lack of tandem or quantitative MS analysis for accurate identification and quantitation of the protein peaks. Also, utilizing tissue lysates, after homogenization, for assessing proteome profiles was not feasible in our study and so, constitutes a further recommendation. Combining proteome patterns with other markers such as CA125 to assess therapeutic response and to increase early detection of relapse of ovarian cancer is a future goal.

In conclusion, MALDI-TOF proteomic profiling has the potential to foster epithelial ovarian cancer diagnosis by facilitating novel protein-based biomarkers’ discovery. MALDI-TOF MS was highly reproducible in detecting ovarian tumor-specific protein profiles, discriminating between early and advanced stages and between serous and non-serous types.
